# Supplementary material for: Clinical Manifestations of Human Exposure to Fungi
Source: J Fungi (Basel). 2023 Mar 21;9(3):381. doi: 10.3390/jof9030381 (PMC10052331; doi:10.3390/jof9030381)
Supplement: Supplementary file 1 [file jof-09-00381-s001.zip › jof-2199613-supplementary.pdf]

Table S1. Fungal allergens approved by the International Allergen Nomenclature Sub-committee (except for a contact exposure for *Trichophyton* spp. and *Malassezia* spp., all allergens are associated with airway exposure) (adapted from http://www.allergen.org/, accessed on 16 January 2023).

Phylum	Species	Allergen	Biochemical name	MW (SDS-PAGE)
Ascomycota	*Alternaria alternata*	Alt a 1		16.4 and 15.3 kDa
		Alt a 3	Heat shock protein 70	
		Alt a 4	Disulfide isomerase	57 kDa
		Alt a 5	Ribosomal protein P2	11 kDa
		Alt a 6	Enolase	45 kDa
		Alt a 7	YCP4 protein	22 kDa
		Alt a 8	Mannitol dehydrogenase	29 kDa
		Alt a 10	Aldehyde dehydrogenase	53 kDa
		Alt a 12	Acid ribosomal protein P1	11 kDa
		Alt a 13	Glutathione-S-transferase	26 kDa
		Alt a 14	Manganese superoxide dismutase	24 kDa
		Alt a 15	Serine protease	58 kDa
	Aspergillus flavus	Asp fl 13	Alkaline serine protease	34 kDa
	Aspergillus fumigatus	Asp f 1	Mitogillin family	18 kDa
		Asp f 2		37 kDa
		Asp f 3	Peroxysomal protein	19 kDa
		Asp f 4		30 kDa
		Asp f 5	Metalloprotease	40 kDa
		Asp f 6	Mn superoxide dismutase	26.5 kDa
		Asp f 7		12 kDa
		Asp f 8	Ribosomal protein P2	11 kDa
		Asp f 9		34 kDa
		Asp f 10	Aspartate protease	34 kDa
		Asp f 11	Peptidyl-prolyl isomerase	24 kDa
		Asp f 12	Heat shock protein P90	90 kDa
		Asp f 13	Alkaline serine protease	34 kDa
		Asp f 15		16 kDa
		Asp f 16		43 kDa
		Asp f 17		27 kDa
		Asp f 18	Vacuolar serine protease	34 kDa
		Asp f 19	HSP 70	69.4 kDa
		Asp f 22	Enolase	46 kDa
		Asp f 23	L3 ribosomal protein	44 kDa
		Asp f 24	EF1B Elongation factor	34 kDa
		Asp f 27	Cyclophilin	18 kDa
		Asp f 28	Thioredoxin	13 kDa
		Asp f 29	Thioredoxin	13 kDa
		Asp f 34	PhiA cell wall protein	20 kDa
		Asp f 35	Cu-Zn Superoxide dismutase similar to Ole e 5	21 kDa
		Asp f 36	Fructose-bisphosphate aldolase	42 kDa
		Asp f 37	Malate dehydrogenase NAD-dependent	35 kDa
		Asp f 38	Uncharacterized Protein	25 kDa
		Asp f 39	FG-GAP repeat protein	35 kDa
	Aspergillus niger	Asp n 14	Beta-xylosidase	105 kDa

	Asp n 18	Vacuolar serine protease	34 kDa
	Asp n 25	3-phytase B	66-100 kDa
Aspergillus oryzae	Asp o 13	Alkaline serine protease	34 kDa
	Asp o 21	TAKA-amylase A	53 kDa
Aspergillus terreus	Asp t 36	Triosephosphate isomerase	28 kDa
Aspergillus versicolor	Asp v 13	Extracellular alkaline serine protease	43 kDa
Candida albicans	Cand a 1	Alcohol dehydrogenase	40 kDa
	Cand a 3	Peroxysomal protein	20 kDa
Candida boidinii	Cand b 2	Peroxisomal membrane protein A	20 kDa
Cladosporium cladosporioides	Cla c 9	Vacuolar serine protease	36 kDa
	Cla c 14	Transaldolase	36.5 kDa
Cladosporium herbarum	Cla h 2		23 kDa
	Cla h 5	Acid ribosomal protein P2	11 kDa
	Cla h 6	Enolase	46 kDa
	Cla h 7	YCP4 protein	22 kDa
	Cla h 8	Mannitol dehydrogenase	28 kDa
	Cla h 9	Vacuolar serine protease	45 kDa
	Cla h 10	Aldehyde dehydrogenase	53 kDa
	Cla h 12	Acid ribosomal protein P1	11 kDa
Curvularia lunata	Cur l 1	Serine protease	31 kDa
	Cur l 2	Enolase	48 kda
	Cur l 3	Cytochrome c	12 kDa
	Cur l 4	Subtilisin like serine protease	54 kDa
Epicoccum purpurascens	Epi p 1	Serine protease	30 kDa
Fusarium culmorum	Fus c 1	Ribosomal protein P2	11 kDa
	Fus c 2	Thioredoxin-like protein	13 kDa
Fusarium proliferatum	Fus p 4	Transaldolase	37.5 kDa
	Fus p 9	Vacuolar serine protease	36.5 kDa
Penicillium brevicompactum	Pen b 13	Alkaline serine protease	33 kDa
	Pen b 26	Acidic ribosomal prot. P1	11 kDa
Penicillium chrysogenum	Pen ch 13	Alkaline serine protease	34 kDa
	Pen ch 18	Vacuolar serine protease	32 kDa
	Pen ch 20	N-acetyl-glucosaminidase	68 kDa
	Pen ch 31	Calreticulin	
	Pen ch 33		16 kDa
	Pen ch 35	Transaldolase	36.5 kDa
Penicillium citrinum	Pen c 3	Peroxysomal membrane protein	18 kDa
	Pen c 13	Alkaline serine protease	33 kDa
	Pen c 19	Heat shock protein P70	70 kDa
	Pen c 22	Enolase	46 kDa
	Pen c 24	elongation factor 1 beta	25 kDa
	Pen c 30	Catalase	97 kDa
	Pen c 32	Pectate lyase	40 kDa
Penicillium crustosum	Pen cr 26	60S acidic ribosomal phosphoprotein P1	11 kDa
Penicillium oxalicum	Pen o 18	Vacuolar serine protease	34 kDa
Stachybotrys chartarum	Sta c 3	Extracellular alkaline Mg-dependent exodesoxyribonuclease	21 kDa
Trichophyton rubrum	Tri r 2	Putative secreted alkaline protease Alp1	29 kDa
	Tri r 4	Serine protease	85 kDa

	Trichophyton tonsurans	Tri t 1		30 kDa
		Tri t 4	Serine protease	83 kDa
	Ulocladium chartarum	Ulo c 1	Alt a 1 homologue	17 kDa
Basidiomycota	*Coprinus comatus*	Cop c 1	Leucine zipper protein	11 kDa
		Cop c 2	Thioredoxin	12 kDa
		Cop c 3		37 kDa
		Cop c 5		16 kDa
		Cop c 7		16 kDa
	Malassezia furfur	Mala f 2	Peroxysomal membrane protein	21 kDa
		Mala f 3	Peroxysomal membrane protein	20 kDa
		Mala f 4	Mitochondrial malate dehydrogenase	35 kDa
	Malassezia sympodialis	Mala s 1		37 kDa
		Mala s 5		18 kDa
		Mala s 6	Cyclophilin	17 kDa
		Mala s 7		16 kDa
		Mala s 8		19 kDa
		Mala s 9		36.7 kDa
		Mala s 10	heat shock protein 70	86 kDa
		Mala s 11	manganese superoxide dismutase	23 kDa
		Mala s 12	glucose-methanol-choline (GMC) oxidoreductase	67 kDa
		Mala s 13	Thioredoxin	13 kDa
	Psilocybe cubensis	Psi c 1		
		Psi c 2	Cyclophilin	16 kDa
	Rhodotorula mucilaginosa	Rho m 1	Enolase	47 kDa
		Rho m 2	vacuolar serine protease	31 kDa
	Schizophyllum commune	Sch c 1	Glucoamylase	61 kDa
Zygomycota	*Rhizopus oryzae*	Rhi o 1	Aspartyl endopeptidase	44 kDa
		Rhi o 2	Cyclophilin	18 kDa

Table S2. Overview of the most common fungal infections.

Type	Infection	Etiological agent	Risk factors	Transmission	Clinical manifestations	Diagnosis	Treatment
Superficial infections	Dermatophytosis	Dermatophytes	Age<br>Gender<br>Other medical conditions	Transfer through shared surfaces and equipment<br>Contact with animals<br>Fungal persistence in household environment	Alopecia<br>Desquamation<br>Edema<br>Erythema<br>Pruritus<br>Purulent exudate	Medical and travel history<br>Symptoms<br>Physical examinations<br>Lab tests	Antifungal medication (azoles, allylamine, epigallocatechin 3-O-gallate)<br>Plant essential oils<br>Nail removal
	Oral candidiasis	*Candida* spp.	Age<br>Gender<br>Medication<br>Other medical conditions	Direct or indirect contact with contaminated people, objects, or surfaces	Ageusia<br>Angular cheilitis<br>Bleeding<br>Denture stomatitis<br>Edema<br>Erythema<br>Leukoplakia<br>Paresthesia<br>Pruritus<br>Thrush<br>Xerostomia	Medical history<br>Symptoms<br>Physical examinations<br>Lab tests	Antifungal medication (azoles)<br>Topic medication (nystatin)<br>Plant essential oils<br>Phytochemicals
	Vaginal candidiasis	*Candida* spp.	Medication<br>Other medical conditions<br>Behavioral aspects	Direct or indirect contact with contaminated people or objects	Edema<br>Erythema<br>Dyspareunia<br>Dysuri<br>Odorless vaginal discharge with a cottage cheese appearance<br>Paresthesia<br>Pruritus<br>Vulvodynia	Medical history<br>Symptoms<br>Physical examinations<br>Lab tests	Antifungal medication (azoles)<br>Topic medication (boric acid, nystatin, flucytosine)<br>Other medications (ibrexafungerp, spilanthol, atorvastatin, probiotics, p-Coumaric acid)
	Candida balanitis	*C. albicans*✦✦✦<br>*C. krusei*	Age<br>Presence of foreskin<br>Medications<br>Other medical conditions<br>Behavioral aspects	Direct or indirect contact with contaminated people or objects	Edema<br>Dysuria<br>Inflammation<br>Phimosis<br>Pruritus<br>Smegma<br>Soreness<br>Tight, shiny skin on the glans	Medical history<br>Symptoms<br>Physical examinations<br>Lab tests	Antifungal medication (azoles)<br>Topic medication (azoles, nystatin)<br>Circumcision
	Pityriasis versicolor	*Malassezia* spp.	Other medical conditions<br>Behavioral aspects<br>Environmental exposure	Outgrows of natural skin populations	Hyperpigmented and hypopigmented macules<br>Pruritus<br>Scaling	Medical history<br>Symptoms<br>Physical examinations<br>Lab tests	Antifungal medication (azoles, terbinafine)<br>Topical medication (pyrithione zinc, selenium sulfide,

							sulfur associated with salicylic acid)
Subcutaneous infections	Eumycetoma	*M. mycetomatis* ✦✦<br>*M. grisea*✦✦<br>*P. boydii*✦✦<br>*L. senegalensis* ✦✦<br>*C. lunata* ✦✦<br>*N. rosatii* ✦✦<br>*Acremonium spp.* ✦✦<br>*Fusarium spp.* ✦✦	Age<br>Gender<br>Other medical conditions<br>Environmental exposure	Fungal spores enter the body through skin injuries<br>No person-to-person transmission	Painless nodules<br>Limb deformation<br>Destruction of muscular and osseous tissue	Medical and travel history<br>Symptoms<br>Physical examinations<br>Lab tests<br>Imagological tests	Antifungal medication (azole)<br>Plant essential oils<br>Surgery<br>Amputation
	Chromoblastomycosis	*Fonsecaea* spp.<br>*Cladophialophora* spp.<br>*P. verrucosa*<br>*Rhinocladiella* spp.<br>*C. ludoviensis*	Age<br>Gender<br>Other medical conditions<br>Environmental exposure	Fungal spores enter the body through skin injuries	Moriform bodies	Medical and travel history<br>Symptoms<br>Physical examinations<br>Lab tests	Antifungal medication (azoles)<br>Surgery<br>Cryotherapy<br>Thermotherapy<br>CO_2 laser<br>Photodynamic therapy
Systemic infections	Aspergillosis<br><br><br><br><br>Aspergilloma	*A. fumigatus*✦✦✦<br>*A. niger*<br>*A. terreus*<br>*A. nidulans*<br>*A. oryzae*<br>*A. fumigatus* ✦✦✦	Medications<br>Other medical conditions<br>Behavioral factors	Inhalation of spores<br>No person-to-person transmission	Arthralgia<br>Cephalalgia<br>Chills<br>Chest pain<br>Cough with hemoptysis<br>Dry cough<br>Dyspnea<br>Eye symptoms<br>Fatigue<br>Fever<br>Skin lesions<br>Weight loss<br>Wheezing	Medical history<br>Symptoms<br>Physical examinations<br>Lab test<br>Imagological test	Oral corticosteroids<br>Antifungal medication (polyenes, azoles, echinocandins)<br>Surgery<br>Embolization
	Candidemia and invasive candidiasis	*Candida* spp.	Medication<br>Other medical conditions<br>Long hospital stays<br>Abdominal surgery<br>Implanted medical devices	Candida gets into the bloodstream, causing candidemia<br>Healthcare-associated infection.<br>No person-to-person transmission.	Abdominal pain<br>Cephalalgia<br>Chills<br>Fatigue<br>Fever<br>Light sensitivity<br>Low blood pressure<br>Memory loss<br>Mental confusion<br>Myalgia<br>Skin rash<br>Vision changes	Medical history<br>Symptoms<br>Physical examinations<br>Lab tests	Antifungal medication (polyenes, azoles, echinocandins, triterpenoids)
	Cryptococcosis	*C. neoformans*✦✦✦<br>*C. gattii*	Age<br>Medication<br>Other medical conditions<br>Behavioral factors<br>Environmental exposure	Inhalation of spores<br>No person-to-person transmission	Behavioral changes<br>Cephalalgia<br>Cervicalgia<br>Chest pain<br>Chills	Medical history<br>Symptoms<br>Physical examinations<br>Lab tests	Antifungal medication (polyenes, azoles)

				Cough<br>Cough with hemoptysis<br>Dyspnea<br>Fever<br>Malaise<br>Mental confusion<br>Nausea and vomiting<br>Sensitivity to light<br>Sleep hyperhidrosis<br>Weight loss	Imagological test	
Blastomycosis	*Blastomyces* spp.	Age<br>Gender<br>Other medical conditions<br>Environmental exposure	Inhalation of spores<br>No person-to-person transmission	Anorexia<br>Chest pain<br>Chills<br>Fever<br>Myalgia<br>Sleep hyperhidrosis<br>Dyspnea<br>Cough<br>Cough with hemoptysis<br>Fatigue<br>Weight loss	Medical and travel history<br>Symptoms<br>Physical examinations, Lab tests<br>Imagological tests	Antifungal medication (polyenes, azoles)
Coccidioidomycosis	*C. immitis* ✦✦<br>*C. posadasii*	Age<br>Race<br>Medication<br>Other medical conditions<br>Environmental exposure	Inhalation of spores<br>No person-to-person transmission	Arthralgia<br>Blood-tinged sputum<br>Cephalalgia<br>Chest pain<br>Chills<br>Cough<br>Dyspnea<br>Fatigue<br>Fever<br>Myalgia<br>Nodules in the lungs<br>Red and spotty rash<br>Sleep hyperhidrosis<br>Weight loss	Medical history<br>Symptoms<br>Physical examinations<br>Lab tests	Antifungal medication (polyenes, azoles, echinocandins, triterpenoids)
Histoplasmosis	*H. capsulatum* ✦✦	Age<br>Medication<br>Other medical conditions<br>Environmental exposure	Inhalation of spores<br>No person-to-person transmission	Arthralgia<br>Cephalalgia<br>Chest pain<br>Chills<br>Cough<br>Fatigue<br>Fever<br>Myalgia	Medical history<br>Symptoms<br>Physical examinations<br>Lab tests<br>Imagological test	Antifungal medication (polyenes, azoles)
Emergomycosis	*Emergomyces* spp.	Medication<br>Other medical conditions	Inhalation of spores	Anemia<br>Ataxia<br>Behavioral changes<br>Cephalalgia	Medical history<br>Symptoms<br>Physical examinations<br>Lab tests	Antifungal medication (polyenes, azoles, echinocandins)

				Crusted hyperkeratotic plaques<br>Endocervical masses<br>Epistaxis<br>Erythema<br>Lobar atelectasis<br>Mental Confusion<br>Nasal congestion<br>Oroantral fistula<br>Pneumonia<br>Seizure<br>Skin lesions<br>Thrombocytopenia<br>Vision changes		
Paracoccidioidomycosis	*Paracoccidioides* spp. ✦	Age<br>Gender<br>Race<br>Other medical conditions<br>Behavioral factors	Inhalation of spores<br>Fungal spores enter the body through skin injuries<br>No person-to-person transmission	Cough<br>Dyspnea<br>Fatigue<br>Fever<br>Hepatosplenomegaly<br>Lymphadenitis<br>Mouth and throat lesions<br>Weight loss	Medical history<br>Symptoms<br>Physical examinations<br>Lab tests<br>Imagological tests	Antifungal medication (polyenes, azoles)
Talaromycosis*	*T. marneffei* ✦✦	Other medical conditions	Inhalation of spores<br>Fungal spores enter the body through skin injuries	Agitation<br>Anemia<br>Confusion<br>Depressed consciousness<br>Dyspnea<br>Fatigue<br>Fever<br>Hepatosplenomegaly<br>Lymphadenopathy<br>Respiratory and gastrointestinal abnormalities<br>Skin lesions<br>Splenomegaly<br>Weight loss	Medical history<br>Symptoms<br>Physical examinations<br>Lab tests	Antifungal medication (polyenes, azoles)
Fusariosis	*Fusarium* spp. ✦✦	Other medical conditions	Fungal spores enter the body through skin injuries	Fever<br>Myalgia<br>Pneumonia<br>Skin lesions<br>Superficial infection in the feet with lymphangitis<br>Toxemic appearance	Medical history<br>Symptoms<br>Physical examinations<br>Lab tests	Antifungal medication (polyenes, azoles)
Hyalohyphomycosis*	Hyaline fungi	Medication<br>Other medical conditions<br>Abdominal surgery<br>Implanted medical devices	Inhalation of spores<br>Fungal spores enter the body through skin injuries	Anemia<br>Arthralgia<br>Cellulitis<br>Cerebritis	Medical history<br>Symptoms<br>Physical examinations	Antifungal medication (polyenes, azoles, echinocandins)

		Trauma<br>Burns<br>Environmental exposure	Ingestion of contaminated food products	Mental confusion<br>Cough<br>Endophthalmitis<br>Fever<br>Fungemia<br>Hepatosplenomegaly<br>Keratitis<br>Lymphadenopathy<br>Onychomycosis<br>Osteomyelitis<br>Peripheral edema<br>Peritonitis<br>Pneumonia<br>Renal failure<br>Sinusitis<br>Weight loss	Lab tests	Topic medication (nystatin)<br>Surgery
Lomentosporiosis	*L. prolificans* ✦✦<br>(formerly *S prolificans*)	Medication<br>Other medical conditions<br>Surgery<br>Trauma<br>Behavioral aspect	Inhalation of spores<br>Fungal spores enter the body through skin injuries<br>Near-drowning<br>Nosocomial infection	Arthralgia<br>Brain abscess formation<br>Cough<br>Decreased visual acuity<br>Dyspnea<br>Edema<br>Embolic phenomena<br>Erythema<br>Eye pain Fever<br>Meningitis<br>Meningoencephalitis<br>Pleuritic chest pain<br>Photophobia<br>Visual disturbances	Medical history<br>Symptoms<br>Physical examinations<br>Lab tests	antifungal medicines (polyenes, azoles, echinocandins)<br>Surgery<br>Hyperbaric chamber
Mucormycosis*	Mucorales ✦✦	Medication<br>Other medical conditions<br>Behavioral factors	Inhalation of spores<br>No person-to-person transmission	Abdominal pain<br>Cephalalgia<br>Chest pain<br>Coma<br>Cough<br>Dyspnea<br>Fever<br>Gastrointestinal bleeding<br>Lesions on nasal bridge or upper inside of mouth<br>Mental status changes<br>Nasal or sinus congestion<br>Nausea and vomiting<br>One-sided facial edema	Medical history<br>Symptoms<br>Physical examinations<br>Lab tests<br>Imagological tests	Antifungal medication (polyenes, azoles)<br>Surgery
Phaeohyphomycosis*	Dematiaceous fungi	Medication<br>Other medical conditions<br>Behavioral factors	Inhalation of spores<br>Immersion in freshwater	Behavioral changes<br>Cough<br>Dyspnea<br>Fever	Medical history<br>Symptoms<br>Physical examinations	Antifungal medication (polyenes, azoles)

				Gastrointestinal bleeding<br>Mental confusion<br>Nausea and vomiting<br>Seizures<br>Sepsis<br>Skin rashes<br>Ulcers	Lab tests	
Pneumocystis pneumonia	*P. jirovecii* ✦✦	Medication<br>Other medical conditions	Healthcare-associated infection.<br>Person-to-person through the air, mainly by droplets.	Chest pain<br>Chills<br>Dry cough<br>Dyspnea<br>Fatigue<br>Fever Hypoxemia<br>Respiratory failure	Medical history<br>Symptoms<br>Physical examinations<br>Lab tests<br>Imagological tests	Antifungal medication (trimethoprim in association with sulfamethoxazole)
Sporotrichosis*	*Sporothrix* spp.	Age<br>Gender<br>Medication<br>Other medical conditions<br>Behavioral aspects	Fungal spores enter the body through skin injuries<br>Contact with animals<br>No person-to-person transmission	Arthralgia<br>Cephalalgia<br>Chest pain<br>Cough<br>Dyspnea<br>Fever<br>Mental confusion<br>Seizures<br>Skin nodules<br>Weight loss	Medical history<br>Symptoms<br>Physical examinations<br>Lab tests<br>Imagological tests	Antifungal medication (polyenes, azoles, supersaturated potassium iodide, terbinafine)

Legend: BAL=broncho alveolar lavage; BMA= bone marrow aspirate; COPD=chronic obstructive pulmonary disease; CT scan=computerized tomography scan; LNA=lymph node aspirate; *fungal diseases with both subcutaneous and invasive presentations. According to the Fungal Priority Pathogen List [141], fungi are signaled as critical (✦✦✦), high (✦✦) and medium priority species (✦).
